# Jak Inhibitors Modulate Production of Replication-Competent Zika Virus in Human Hofbauer, Trophoblasts, and Neuroblastoma cells

**DOI:** 10.20411/pai.v2i2.190

**Published:** 2017-05-24

**Authors:** Christina Gavegnano, Leda C. Bassit, Bryan D. Cox, Hui-Mien Hsiao, Erica L. Johnson, Mehul Suthar, Rana Chakraborty, Raymond F. Schinazi

**Affiliations:** 1 Center for AIDS Research, Laboratory of Biochemical Pharmacology, Department of Pediatrics, Emory University, Atlanta, Georgia; 2 Emory Vaccine Center, Yerkes National Primate Center, Emory University, Atlanta, Georgia

**Keywords:** Zika, mother-to-child-transmission (MTCT), placenta, Hofbauer, trophoblasts, HIV, interferon, Jak-STAT, activation, ruxolitinib, CNS

## Abstract

Zika Virus (ZIKV) is a flavivirus that has been implicated in causing brain deformations, birth defects, and microcephaly in fetuses, and associated with Guillain-Barre syndrome. Mechanisms responsible for transmission of ZIKV across the placenta to the fetus are incompletely understood. Herein, we define key events modulating infection in clinically relevant cells, including primary placental macrophages (human Hofbauer cells; HC), trophoblasts, and neuroblastoma cells. Consistent with previous findings, HC and trophoblasts are permissive to ZIKV infection. Decrease of interferon signaling by Jak ½ inhibition (using ruxolitinib) significantly increased ZIKV replication in HC, trophoblasts, and neuroblasts. Enhanced ZIKV production in ruxolitinib-treated HC was associated with increased expression of HLA-DR and DC-SIGN. Nucleoside analogs blocked ruxolitinib-mediated production of extracellular virus. Although low-level ZIKV infection occurred in untreated HC and trophoblasts, replicating virions were incapable of infecting naive Vero cells. These deficient virions from untreated HC have “thin-coats” suggesting an immature structure. Blocking Jak ½ signaling (with ruxolitinib) restored replication competence as virions produced under these conditions confer cytopathic effects to naive Vero cells. These data demonstrate that Jak-STAT signaling directly impacts the ability of primary placental cells to produce replication-competent virus and is a key determinant in the production of mature virions in clinically relevant cells, including HC and trophoblasts. Design of targeted agents to prevent ZIKV replication in the placenta should consider Jak ½ signaling, the impact of its block on ZIKV infection, and subsequent transmission to the fetus.

## INTRODUCTION

The family *Flaviviridae* encompasses over 70 members, including West Nile Virus (WNV), Dengue Virus (DENV), Japanese Encephalitis Virus (JEV), Yellow Fever Virus (YFV), and Zika Virus (ZIKV) [1]. These mosquito-transmitted viruses can cause hemorrhagic fever, encephalitis, or serious CNS defects [2–6]. The ZIKV has been found in various compartments within the CNS, including the brain and cerebrospinal fluid of fetuses at autopsy, whose mothers were infected during pregnancy with ZIKV [2–6]. Associated brain abnormalities include microcephaly with reduced viability and cell growth in human neurospheres and brain organoids [5, 7]. Together, these data underscore the fact that mother-to-child transmission (MTCT) of ZIKV represents a major health concern.

The mechanisms of immune evasion, establishment of infection across target cells, and the relationship between these dynamics and transmission of ZIKV across the placenta to the fetus are poorly understood. Previous studies have demonstrated that flaviviruses employ a variety of host immune evasion strategies to establish primary infection including virus-modulated blockade of the antiviral interferon α/β (IFN-α/β) response [8–12]. The antiviral IFN response is a key modulator of innate immunity, orchestrating a first line of defense to facilitate paracrine and autocrine production of IFN-α/β and priming of interferon signaling genes (ISG). These genes crosstalk with bystander cells to promote expression of genes that perform antiviral functions. The IFN-based signaling can also prime, recruit, and activate phagocytic macrophages, providing a second layer of innate immune function [10, 13]. Since IFNs represent a key and early antiviral modulator in innate immunity triggered by viral infection, flaviviruses have evolved strategies to block early innate immune signaling in order to establish infection [1, 9, 14, 15].

An important mechanism for flaviviral blockade of IFN production is direct interference with the Janus Kinase Signal Transducer and Activation of Transcription (Jak-STAT) pathway [9, 12, 15]. Recent reports demonstrate a direct link between Jak-STAT antagonism by ZIKV, blockade of the IFN-α/β antiviral response, and evasion of the innate immune response that allows the virus to establish replication undetected [16]. Activation of the Jak-STAT pathway is a major mechanism responsible for indirect or direct autocrine and paracrine modulation, followed by signaling that results in production of IFN-α/β, which in turn facilitates a rapid paracrine and autocrine production of this cytokine, thereby triggering an antiviral milieu [8, 10]. It has been reported that various structural proteins of WNV, DENV, JEV, and other flaviviruses directly prevent activation of the Jak-STAT pathway, including NS5, NS2A/B, and NS4A/B proteins which impact Tyk2 and STAT2, and can promote ubiquitination of the kinases necessary to phosphorylate Jaks and STATs [17]. Phosphorylation of STAT is necessary for nuclear translocation of the STATs, which in turn bind to specific transcription sites and promote production of IFN-α/β and other antiviral cytokines [17, 18]. Previous studies have demonstrated that the immunological milieu within the placenta, and specifically in placental macrophages (Hofbauer cells, HC), could serve as a protective barrier to mother-to-child transmission (MTCT) of HIV-1 or other viral infections [19]. It has also been reported that increased activation or proliferation promoted by pro-inflammatory cytokines can significantly increase the amount of virus that is produced by infected macrophages and macrophage-like cells [20, 21]. Therefore, activation and inflammation that is present in the mother as a function of primary ZIKV infection may directly or indirectly impact viral production in macrophage-like cells. Increased viral replication may impact whether the virus can efficiently cross the placenta to establish infection in the fetus. Understanding these events, their role in establishment of infection in infected mothers, and subsequent translation to an immunological milieu that may promote transmission of the virus across the placenta is critical to designing specific, safe inhibitors to block key events in ZIKV infection and MTCT.

Ruxolitinib is a selective Jak ½ inhibitor that is FDA approved for treatment of myelofibrosis and polycythemia vera. (Jakafi package insert, [22]). Ruxolitinib demonstrates potent *in vivo* inhibition of circulating plasma IFN-α/β, TNF-α, IL-1-α/β, IL-6, C reactive protein (CRP), and D-dimer as a direct function of its potent, selective Jak ½ blockade [22]. Since the Jak-STAT pathway is a key modulator of the interface between host immunity and viral infection [18], we sought to use supraphysiological concentrations of ruxolitinib as a tool to understand the blockade of Jak-STAT signaling and subsequent production of ZIKV infection in clinically relevant cells including primary human HC, trophoblasts, and neuroblastoma cells.

## MATERIALS AND METHODS

### Ethics Statement

Term placentae (> 37 weeks' gestation) from 12 HIV-1, rapid plasma regain, and hepatitis B serum-negative women were obtained immediately following elective caesarian section without labor from Grady Memorial and Emory Midtown Hospitals in Atlanta, GA. All patients gave written informed consent. Approval of the study was granted from Emory University Institutional Review Board (IRB) and Grady Research Oversight Committee. Peripheral blood was obtained from healthy adult volunteer donors according to a protocol approved by the Emory University IRB. Written informed consent was obtained from donors, and the identity of samples was removed prior to handling by laboratory personnel.

### Source of antiviral agents

2′-C-Methylcytidine (2′-C-MeC) and β-D-*N*^4^-hydroxycytidine (NHC) were synthesized in our laboratory and were found by liquid chromatography and mass spectroscopy to be more than 98% pure.

### Source of virus

Virus stock of PRVABC59 (NCBI accession KU501215) was used to generate virus for all studies. Briefly, virus was expanded in Vero cells in T-75 culture flasks in 2% FBS-containing MEM medium for 72 hours prior to harvest of the virus, followed by centrifugation, filtration, and titration of stocks in baby hamster kidney (BHK) cells by plaque forming assay.

### Isolation and culture of cells

Primary HC and trophoblasts were dissected from membrane-free villous placenta, as previously described [19]. The HC were isolated and purified with magnetically labeled anti-CD14 microbeads (positive selection) according to the manufacturer's instructions (Miltenyi Biotec, San Diego, CA). Trophoblasts were isolated and purified by negative selection with magnetic beads (Miltenyi Biotec, San Diego, CA). Cells were cultured and maintained as previously described [19]. Human neuroblastoma cells (U251) were obtained from Sigma Aldrich (09063001; St. Louis, MO) and cultured according to the manufacturer's instructions. Vero or BHK cells were obtained from ATCC (Manassas, Virginia) and cultured according to the manufacturer's protocol in MEM or DMEM medium supplemented with 10% fetal bovine serum (FBS; Atlanta Biologicals, Lawrenceville, GA).

### ZIKV Infections

The HC or trophoblasts were seeded at 7 x 10^4^ cells/well in 96-well microplates (Corning/Cell Star Inc., St. Louis, MO), and allowed to rest for 24 hours at 37°C prior to use for infections. After 24 hours at rest, cells were treated with 0.1, 1.0, or 10 μM ruxolitinib for 24 hours prior to infection, or were maintained in ruxolitinib-free medium (for use with infections in the absence of ruxolitinib). For infections with ruxolitinib, the drug was maintained in the culture for the duration of the infection and culture. The HC or trophoblasts were infected with an MOI of 1 of Puerto Rican strain (PRVABC59) for 2 hours prior to removal of virus and culture with or without 0.1, 1.0, or 10 μM of ruxolitinib. Cells and supernatants were harvested on day 6 after infection for real time RT-PCR (cells), flow cytometry (cells), supernatant transfer (virally induced cytopathic effect [CPE]) assays (supernatants), or cryo-electron microscopy (EM). Day 6 was selected because previous reports demonstrated that ≥ day 4 post-infection demonstrated peak and robust ZIKV replication. This kinetic was confirmed in our group (data not shown).

The U251 human neuroblastoma cells (ATCC) were seeded at 2 x 10^4^ cells/well in 24-well plates and treated with or without 0.1, 1, or 10 μM ruxolitinib for 24 hours prior to infections with an MOI of 1 of PRVABC59 for 2 hours. After 2 hours, the virus was removed and cells were cultured with or without 0.1, 1, or 10 μM of ruxolitinib for 4 days prior to quantification of viral induced CPE or ZIKV infected cells using flow cytometry.

### Supernatant transfer assays

Vero cells were seeded at 1 x 10^4^ cells/well in MEM medium (Thermo Fisher, Waltham, MA) supplemented with 2% FBS in 96-well plates. Supernatants (100 μL) from HC and trophoblast cells infected with ZIKV and harvested on day 6 (described above) were added to Vero cell cultures. After 4 days in culture (37°C, 5% CO_2_), the virally induced CPE of the supernatants was quantified using the CellTiter 96^®^ AQueous One Solution Cell Proliferation kit (Promega, Madison, WI), which uses a tetrazolium-based dye (3-(4,5-dimethyl-2-yl)-5-(3-carboxymethoxyphenyl)-2-(4-sulfophenyl)-2H-tetrazolium and an electron coupling reagent (phenazine ethosulfate) to quantify living cells. This assay measures the ability of extracellular virus produced by HC or trophoblast cells by quantifying the amount of viral-induced CPE conferred in permissive Vero cells.

### Real-time reverse transcription PCR

The HC or trophoblasts were infected in the presence or absence of ruxolitinib as described previously and cells were harvested on day 6. Then RNA was reverse transcribed into cDNA and amplified in a one-step RT-PCR multiplex reaction with the LightCycler 480 RNA Master Hydrolysis Probe (Roche, Indianapolis, IN) using highly conserved sequences complementary to a 76 bp fragment from the ZIKV envelope gene as previously described [23] and an endogenous control (TaqMan Ribosomal RNA Control or beta globin Reagents; Applied Biosystems, Foster City, CA). The procedure was performed using the LightCycler 480 Instrument II (Roche). The analysis was performed by using the sample crossing point (Cp) value, which is the point at which an amplified product (the fluorescence of a sample rises above the background fluorescence) is visible. Relative quantification was determined using the ZIKV RNA Cp values relative to infected/untreated controls.

### Flow Cytometry

The HC, trophoblasts, or U251 cells were infected as described above. Cells were fixed using CytoFix/Cytoperm and permeabilized according to the manufacturer's protocol (BD Biosciences, San Jose, CA), followed by staining with pan flavivirus 4G2 antibody (Millipore, Billerica, MA) and secondary staining with goat anti-mouse 488 (BD Biosciences). The HC were also stained with HLA-DR-VioBlue (Miltenyi Biotec) and DC-SIGN-APC (BD Biosciences), and 4G2^+^ cells, together with HLA-DR^+^ and DC-SIGN^+^ cells were quantified with flow cytometry (MacsQuant, Miltenyi Biotec). FACS plots were established based on forward and side scatter, doublet discrimination, and gates were set based on uninfected, stained control cell samples. Analysis was performed with MacsQuantify software (Miltenyi Biotec).

### Cryo-electron Microscopy

Supernatant samples were treated with 4% paraformaldehyde for 24 hours at 4°C using a 1:1 ratio of paraformaldehyde to supernatant. Particles were deposited onto a charged carbon grid for 10 minutes and washed 3 times with deionized water. The samples were stained using uranyl acetate solution for 30 minutes and vacuum dried. Negative stain images were collected on a JEOL JEM-1400 transmission electron microscope operating at 120 kV and processed with IMOD software.

## RESULTS

### ZIKV infections in HC or trophoblasts with or without ruxolitinib:

Cells were treated with 0.1, 1.0, or 10 μM ruxolitinib for 24 hours prior to infection in the presence or absence of ruxolitinib with an MOI of 1 of PRVABC59. Virus was removed after 2 hours and cells were cultured in the presence or absence of ruxolitinib. Cells and supernatants were collected on day 6 post-infection. Cells were used for RT-PCR to quantify ZIKV RNA replication intracellularly.

**Figure 1.**
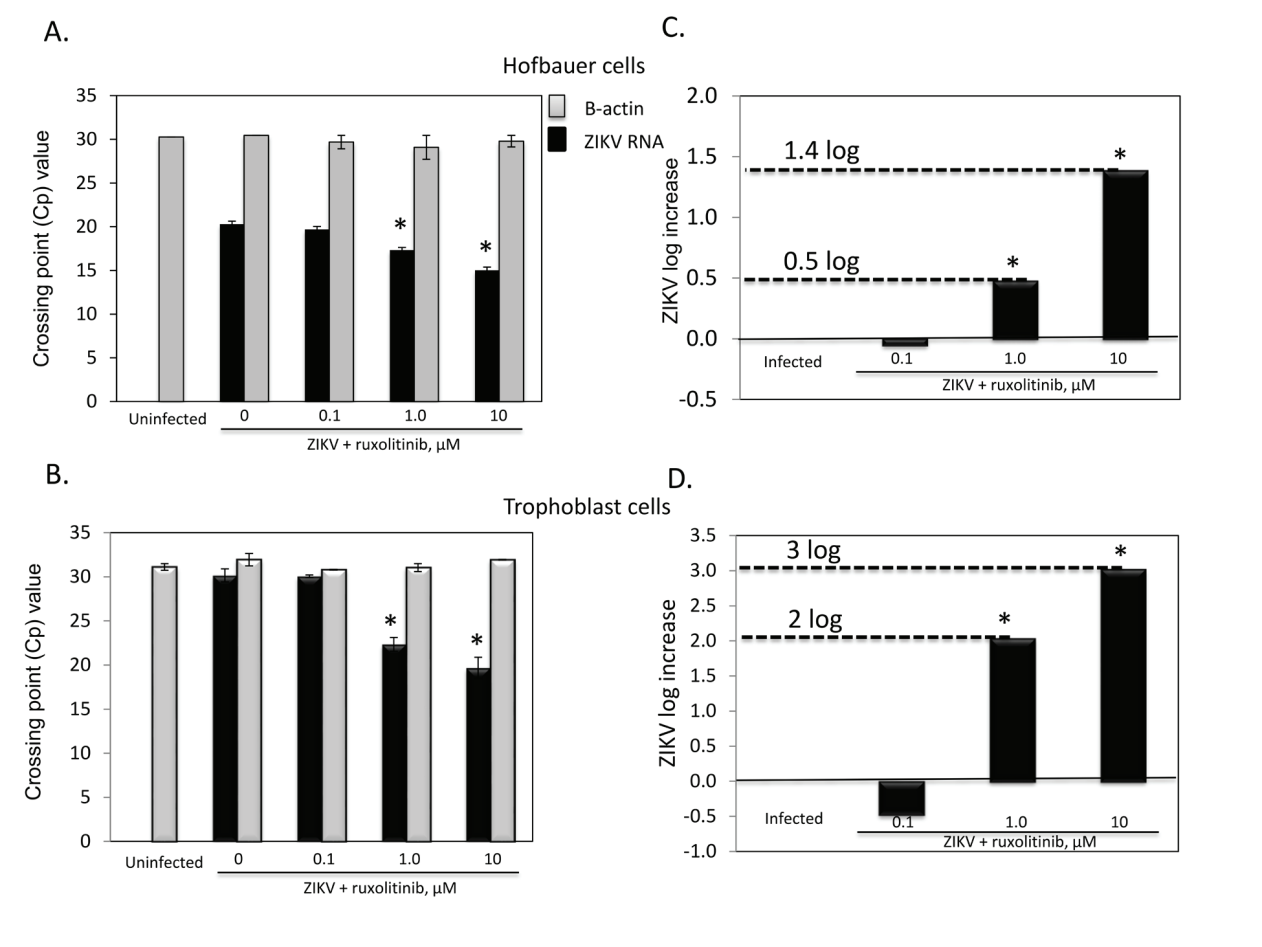
**Effect of Jak½ blockade with ruxolitinib on permissiveness of primary human Hofbauer or trophoblast cells to ZIKV PRVABC59.** Primary human Hofbauer cells (HC) or trophoblasts were permissive to PRVABC59 MOI 1 (A, B respectively; ZIKV RNA RT-PCR). The analysis was performed by using the sample crossing point (Cp) value which is the point at which an amplified product (fluorescence of a sample rises above the background fluorescence) is visible. Relative quantification was determined using the ZIKV RNA Cp values relative to infected/untreated controls. One or 10 μM ruxolitinib significantly increased virus replication versus infected/untreated controls in both cell types (A, B); * *P* < 0.01, One way ANOVA. Beta actin controls demonstrated that total cell numbers were not reduced across conditions and compared to uninfected controls (A, B, grey bars b-actin internal control, panels A, B). Ruxolitinib results in 0.5 to 1.4 log increase of ZIKV RNA replication in HC (C), and 2 to 3 log increase in trophoblasts (D); * *P* < 0.01, One Way ANOVA versus infected/untreated control. Infected cells were normalized to zero but had a crossing point value of 20 and 30 respectively for HC (A) and trophoblasts (B). Results were also normalized with endogenous beta actin controls.

Primary human HC or trophoblasts are permissive to PRVABC59 (Figures 1A, B). In addition, 1 or 10 μM Ruxolitinib significantly increased intracellular virus replication compared to infected/untreated controls, with 0.5 to 1.4 log increase in ZIKV replication in HC, and 2 to 3 log increase in trophoblasts (Figures 1C, D). The beta globin control demonstrated that total cell numbers were not reduced across conditions compared to uninfected controls of both cell types (Figures 1A, B, grey bars, panels A, B). Infected/untreated cells were normalized to calculate log increase in ZIKV replication in the presence of ruxolitinib. Results were also normalized with endogenous beta globin controls.

### Effect of ZIKV infection alone or in the presence of Jak 1/2 blockade with PRVABC59 on activation markers in primary HC:

**Figure 2.**
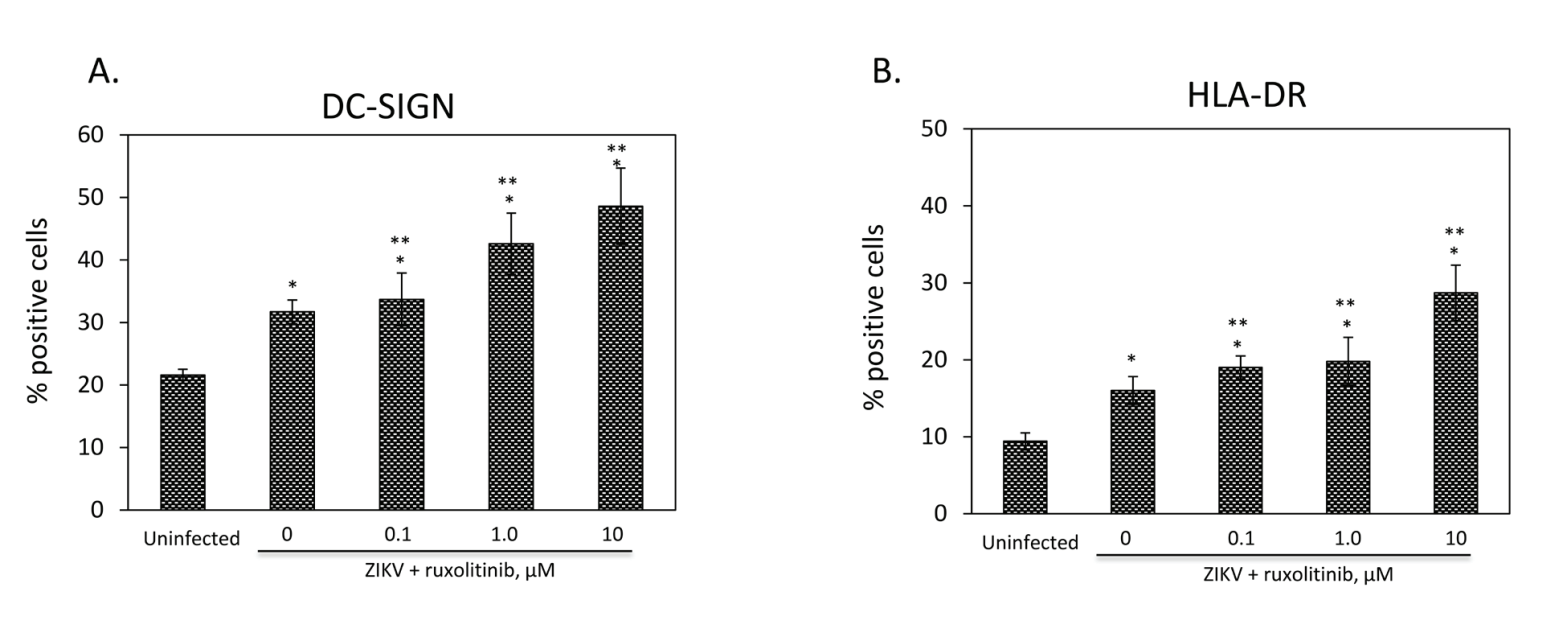
**Effect of ZIKV infection alone or in the presence of Jak½ blockade PRVABC59 on activation markers in primary human hofbauer cells.** ZIKV (MOI 1) significantly increased (* *P* < 0.01 versus uninfected controls, one way ANOVA; ** *P* < 0.01 one way ANOVA versus no drug controls) DC-SIGN and HLA-DR (A and B, respectively).

Primary HC were treated with 0.1, 1.0, or 10 μM ruxolitinib for 24 hours prior to infection in the presence or absence of ruxolitinib with an MOI of 1 of PRVABC59. Virus was removed after 2 hours and cells were cultured in the presence or absence of ruxolitinib for 6 days prior to extracellular staining with flurochrome-conjugated mAb to DC-SIGN (Figure 2A), or HLA-DR (Figure 2B), and sub-gated on forward and side scatter plots and doublet discrimination. Infection with ZIKV significantly increased DC-SIGN and HLA-DR (Figures 2A and B, respectively).

### Virally induced CPE in Vero cells with supernatant transfers from ZIKV-infected HC or trophoblasts

**Figure 3:**
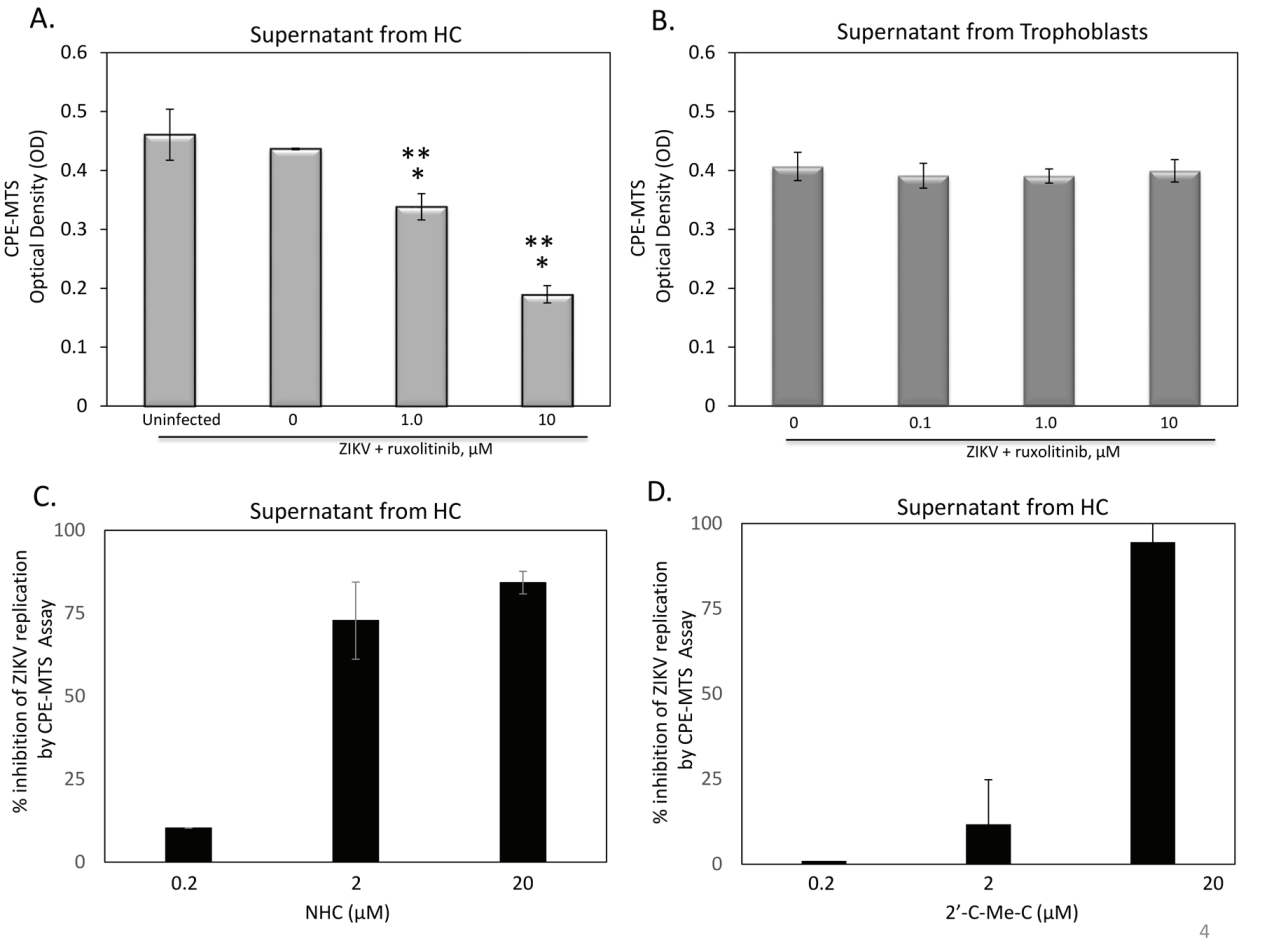
**Effect of ruxolitinib on ability of HC or trophoblast supernatants infected with ZIKV PRVABC59 to confer virally induced CPE in Vero cells.** Primary HC or trophoblasts were infected with MOI 1 of ZIKV PRVABC59 to induce viral production. Cell death (cytopathic effect; CPE) induced by the viral supernatants were quantified with an MTS assay. Supernatants from HC infected with PRVABC59 did not confer virally induced CPE (A), whereas addition of 1 and 10 μM in HC cultures demonstrated a dose dependent increase in virally induced CPE in Vero cells (A). Supernatants from trophoblasts, either in the absence or presence of ruxolitinib did not confer virally induced CPE in Vero cells (B). Antiviral agents β-D-N4-hydroxycytidine (NHC) and 2′-C-methyl-cytidine (2′-Me-C) added in various concentrations to HC or trophoblast cultures also maintained with 1 μM ruxolitinib during ZIKV infections demonstrated a dose dependent protection from virally induced CPE for HC supernatants transferred to Vero cells (C, D). Supernatants for trophoblasts, in contrast, did not confer virally induced CPE for any conditions, therefore effect of antiviral agents could not be measured (data not shown). * demonstrates significant difference versus infections in the absence of ruxolitinib; ** demonstrates significant difference versus uninfected controls (*P* < 0.05, One Way ANOVA).

Consistent with previous findings, primary HC and trophoblasts infected with an MOI of 1 of ZIKV PRVABC59 are permissive for infection and productive virus replication [25]. Cell death (CPE) induced by the viral supernatants was quantified with an MTS cell proliferation assay, demonstrating that the virus produced was replication competent, capable of infecting naive Vero cells, and subsequently conferring a virally induced CPE. Supernatants from HC infected with PRVABC59 did not confer virally induced CPE (Figure 3A), whereas addition of 1 and 10 μM ruxolitinib in HC cultures demonstrated a concentration-dependent increase in virally induced CPE in Vero cells (Figure 3A). It has been previously reported [25] that supernatants from HC infected with ZIKV conferred a donor-dependent increase in focus-forming units for Vero cells infected with supernatants from HC over the course of 72 hours. The replication competence of the virions from HC to confer virally induced CPE, which is indicative of productive replication, was not measured. Additionally, the focus-forming assay as a measure of productive, replication-competent virus would not explain the internalization of defective virions accounting for 4G2^+^ cells. The results of virally-induced CPE (cell death), reported in this study, confirmed that the virus was able to induce cell death, and therefore was replication competent. Therefore, it is possible that differences in assay readout, and what each assay quantifies, account for differences in observed findings with HC supernatants [24, 25]. Supernatants from trophoblasts, either in the absence or presence of ruxolitinib did not confer virally induced CPE in Vero cells (Figure 3B). Antiviral agents β-D-*N*_4-_hydroxycytidine (NHC) or 2′-C-methyl-cytidine (2′-Me-C) added in various concentrations to HC or trophoblast cultures, which were maintained with 1 μM ruxolitinib during ZIKV infection, demonstrated concentration-dependent protection from virally induced CPE from HC supernatants transferred to Vero cells (Figures 3C, D). Supernatants from tropho-blasts, in contrast, did not confer virally-induced CPE under any conditions; therefore the effect of antiviral agents could not be measured (data not shown).

### Antiviral effect of nucleoside analogs measured by transfer of supernatants produced by HC or tropho-blasts to uninfected Vero cells:

Primary HC or trophoblasts were infected with an MOI of 1 of ZIKV PRVABC59 as described in Figure 3 with or without 1 μM ruxolitinib and with or without the antiviral agents 2′-C-Me-C or β-D-*N*_4_-hydroxycytidine (NHC). Supernatants from HC or trophoblasts were transferred to uninfected Vero cells. Virally induced CPE was quantified 4 days after supernatant transfer. Supernatants from HC or trophoblasts in the absence of ruxolitinib did not confer virally-induced CPE in uninfected Vero cells (data not shown). Supernatants from trophoblasts exposed to 1 μM ruxolitinib did not confer virally induced CPE in uninfected Vero cells (data not shown; cells were quantified for up to 6 days, and cultures continued to grow without any induction of CPE, similar to uninfected controls). Supernatants from HC treated with 1 μM ruxolitinib during ZIKV infection induced CPE, and antiviral agents 2′-C-MeC and NHC added to the HC cultures blocked ZIKV-induced CPE in Vero cells (median effective concentration and concentration of drug required to inhibit 90 % of viral replication; EC_50_ 1.7-17.3 μM, EC_90_ 12.8 to approximately 20 μM; Figure 3).

### Jak 1/2 inhibitor ruxolitinib significantly increased ZIKV infection and virally induced CPE in human neuroblastoma cells:

**Figure 4.**
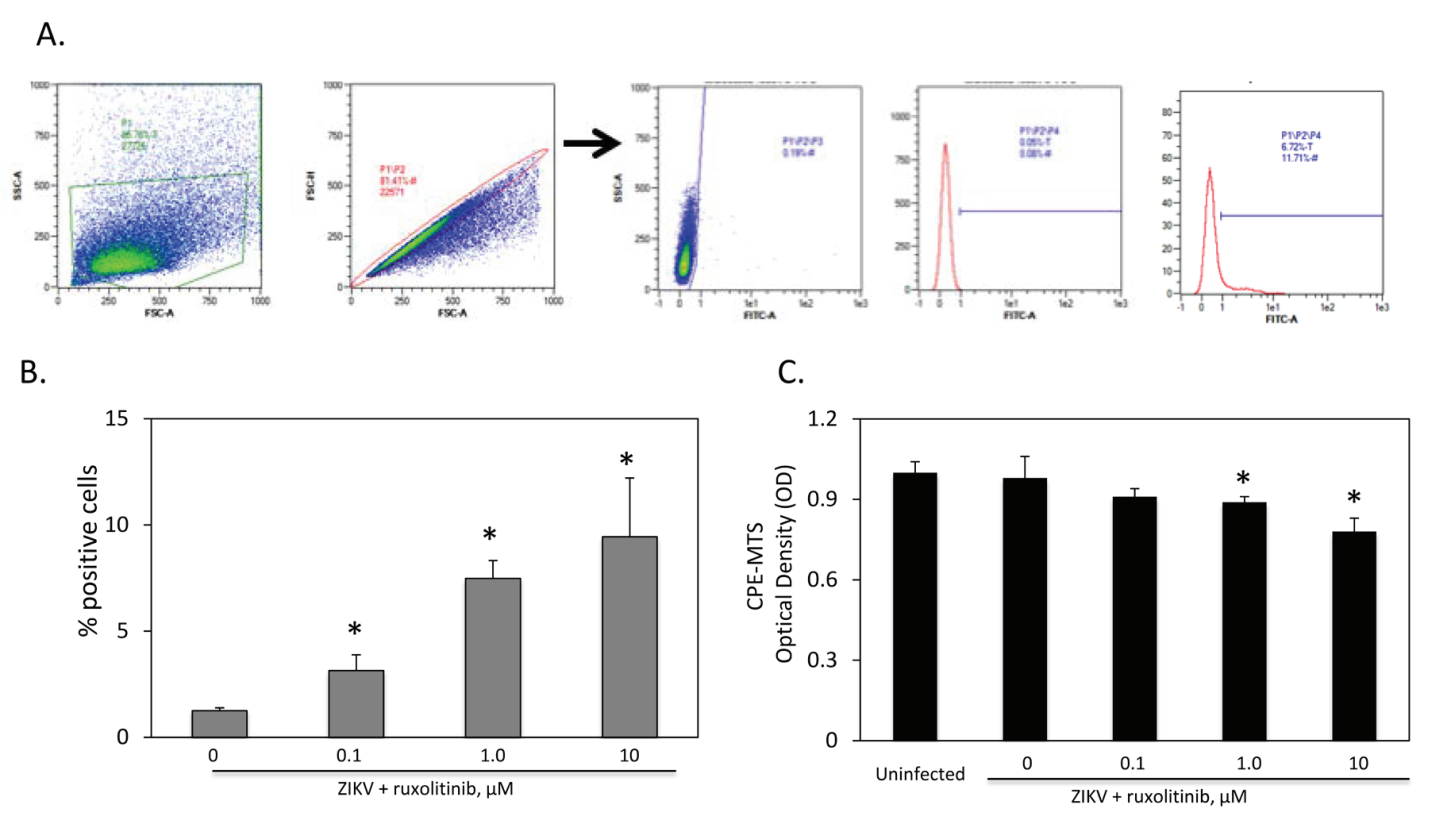
**Jak ½ inhibitor ruxolitinib significantly increases ZIKV+ cells and virally induced CPE in human neuroblastoma cells.** U251 human neuroblastoma cells were infected with MOI 0.5 PRVABC59 for 2 hr +/- 24 hr ruxolitinib pre treatment that was maintained throughout duration of infection. CPE and FACS measured day 5 post infection, after visual monitoring daily for CPE induction. Flow cytometry gating strategy, A; 4G2+ cells, B; Optical Density (OD) values obtained from the MTS assay for virus induced cytopathic effect (CPE) with ZIKV PRVABC59, C. * indicates significant difference versus no drug controls, *P* < 0.05, One way ANOVA.

The U251 human neuroblastoma cells were infected with PRVABC59 at an MOI of 0.5 for 2 hours, with or without 24 hours of ruxolitinib pre-treatment that was maintained throughout the duration of infection. After visual monitoring daily for CPE induction, results for CPE and FACS were measured on day 5 post-infection. The Flow cytometry gating strategy is depicted in Figure 4A. The U251 cells are weakly permissive to ZIKV infection with PRVABC59 (0.5 MOI yields approximately 1.5% to 2.5% 4G2^+^ cells, and ruxolitinib significantly increased the percentage of 4G2^+^ cells, Figure 4B). Optical density values with ZIKV PRVABC59 demonstrated that ZIKV conferred virally induced CPE, and that ruxolitinib conferred a concentration-dependent increase in virally induced CPE (Figure 4C).

### Negative-Staining Electron microscopy of virions produced from HC or trophoblasts in the presence or absence of ruxolitinib compared to Vero cells:

**Figure 5.**
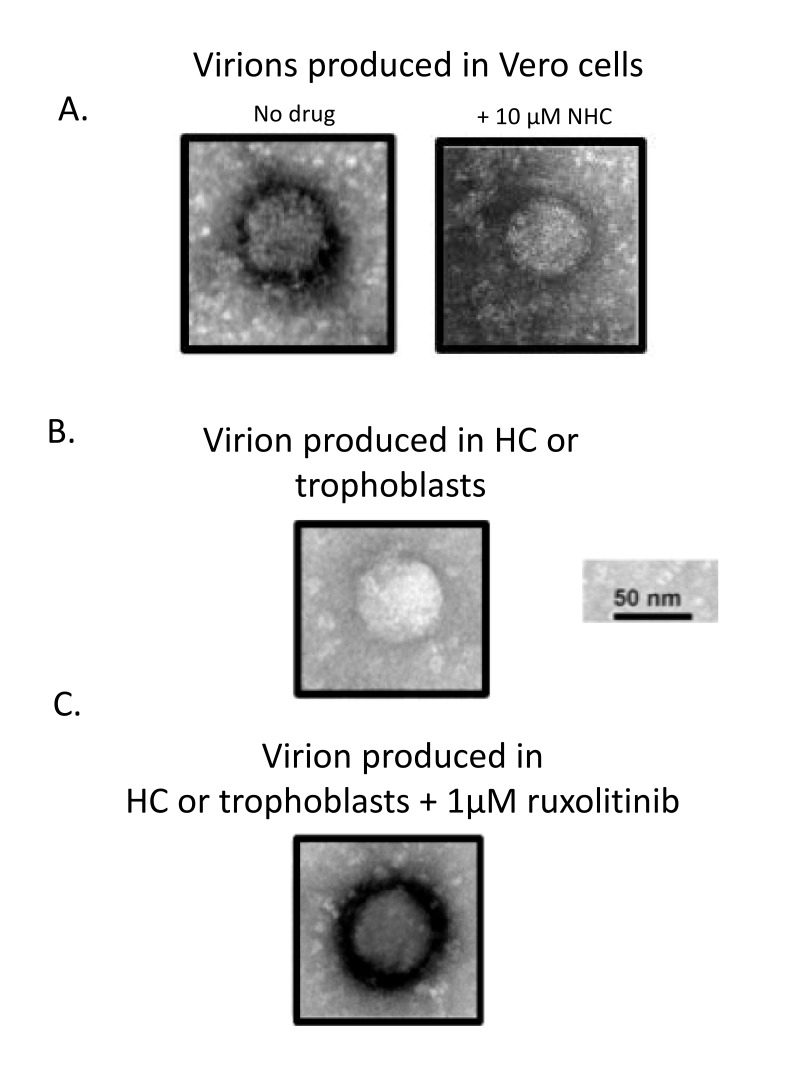
**HC and trophoblasts produce “thin coat” ZIKV but “thick coat” viruses similar to Vero cells in the presence of ruxolitinib.** Supernatants from Vero cells infected with PRVABC59 produce virions with “thick coats” (A; left panel) which were confirmed to be replication competent with supernatant transfer assays. Block of ZIKV replication in Vero cells with 10 μM NHC resulted in reduction of number of extracellular virions, however virions observed were “thick coat” (A; right panel). HC or trophoblasts infected with PRVABC59 produced “thin coat” viruses (representative images; B) whereas HC infected in the presence of 1 μM ruxolitinib produced “thick coat” viruses (representative images; C).

Supernatants from Vero cells infected with PRVABC59 produced virions with “thick coats” (Figure 5A, left panel), which were confirmed to be replication competent with supernatant transfer assays, and have been reported in the literature [26, 27] (data not shown). Blocking ZIKV replication in Vero cells with 10 μM NHC resulted in a reduction of the number of extracellular virions; however, the virions observed were virions with thick coats (Figure 5A, right panel). The HC or trophoblasts infected with PRVABC59 produced “thin coat” viruses (Figure 5B, representative virion), whereas HC or trophoblasts infected in the presence of 1 μM ruxolitinib produced thick coat viruses (Figure 5C, representative virion). Other morphological characteristics included circular shape and size, which was the same for all virions with diameters of approximately 40 to 50 nm; consistent with cryo-EM structures.

### Models of immature versus mature ZIKV virions:

**Figure 6.**
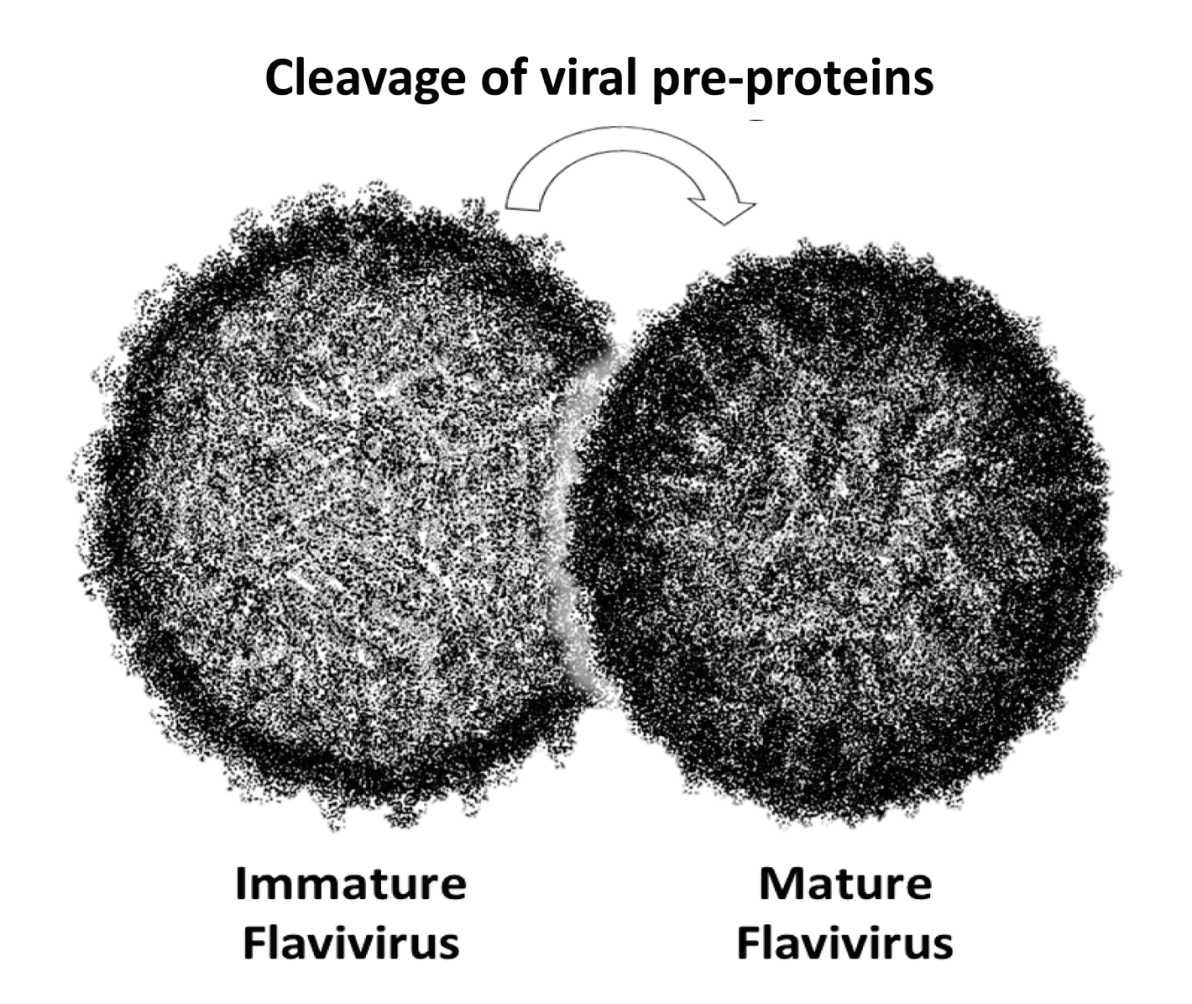
**Model of immature and mature flavivirus.** Predictive modeling based on published crystallographic reports of immature flaviviruses and the mature ZIKV virus protein structure, demonstrating that the visual appearance of immature flavivirus virions is less dense, and contains a “thin coat” versus the dense, “thick coat” of a mature flavivirus. These models are similar to the images collected for HC and trophoblast supernatants +/- ruxolitinib, wherein virions from HC and trophoblasts without ruxolitinib generated immature virions, and virions grown in HC and trophoblasts in the presence of ruxolitinib produced mature virions (Figure 5).

Visual representations of immature and mature flavivirus particles were sought for comparison with the negative-stain electron microscopy results. The mature virion image was generated using the cryo-EM structure of ZIKV produced in Vero cells (PDBID 5IRE, 3.8 Å; Figure 6). No structure exists for immature ZIKV. The cryo-EM structure of low-pH, immature DENV was used as a surrogate because there is high sequential similarity to ZIKV (58% identical) [28] (PDBID 3C6R, 25 Å). Only the backbone (first carbon) Cα positions are displayed for the mature and immature models resulting in a similar number of points in the visuals (approximately 1,400 *versus* 1,700, respectively). The immature flavivirus was characterized by a thin circular band of points while the mature representation exhibits a thick, dense band of points. The mature particle appears more compact than the immature virion (Figure 6).

## DISCUSSION

With the ZIKV epidemic reaching a pandemic in certain parts of South and Central America and the Caribbean, rapid understanding of how the virus replicates across relevant target cells, key events involved in transmission of the virus to the fetus, and potential immunological modulators that govern replication competence are important to determine, but poorly understood. Elucidating the delicate host-pathogen interplay provides a springboard from which to design safe, specific, and direct-acting antiviral as well as immunomodulator-based agents that can block sentinel events involved in viral replication and MTCT. Previous reports by our group demonstrated that primary human HC and trophoblasts can be infected by PRVABC59 ZIKV, and that infection was associated with perturbation of extracellular cytokines that govern innate and adaptive immunity, as well as activation that may in turn modulate MTCT in placental cells [25]. Additionally, a recent report links STAT2 antagonism by ZIKV as a primary mechanism that the virus employs to down-regulate the Jak-STAT-controlled antiviral IFN-α/β response, underscoring the interplay between the Jak-STAT pathway and the establishment of a productive ZIKV infection [16]. Recent reports demonstrated that viral replication coincides with induction of type I interferon (IFN), proinflammatory cytokines including IL-6, IFN-α, MIP-1-α/β, IL-1RA, CXLC8, and MCP-1 [25]. Release of these cytokines was coupled with antiviral gene expression. The relationship of these findings to drug-based initiatives, and the direct role of Jak-STAT signaling in impacting these events requires further characterization.

Therefore, we sought to answer several key questions, which may guide drug-based eradication initiatives: 1) Can HC and trophoblasts become infected with ZIKV? 2) Is infection in HC associated with cellular activation that may impact MTCT? 3) Are immunological components, especially intracellular signaling cascades that modulate IFN-α/β, able to govern permissiveness to infection in clinically relevant cells? 4) Can infection in HC and trophoblasts be inhibited with antiviral agents? 5) Are mechanisms governing permissiveness in HC and trophoblasts also relevant to other cell types such as human neuronal cells? and 6) Do these mechanisms result in production of immature, defective virions in HC and trophoblasts with compromised replication competence in these cells? To carefully answer these questions, all studies with ruxolitinib were performed with 24 hour pre-treatment of cells with the drug prior to exposure to virus. This was done to allow for a complete block of Jak ½ signaling prior to infection, thereby allowing the mechanism of Jak ½ blockade and subsequent downstream effects on viral replication and replication competence to be studied.

Previous reports indicate that ZIKV can infect primary human HC and trophoblasts [25], and emphasized that the infection levels reported in trophoblasts were markedly lower than those observed for HC [25]. Earlier reports stated that ZIKV infection in trophoblasts may be limited due to their production of interferons, which function in a protective antiviral capacity [8]. These data were complemented by the suggestion that indirect interplay between trophoblasts and the virus may in turn modulate a milieu that promotes MTCT [8, 19, 25, 29, 30], as opposed to direct and productive viral replication. Differences in viral strains used (PRVABC59 versu*s* MR766 and FSS13025) as well as the timeframe for terminal harvest and sensitivity of assays used may account for differences in reported results. Although these reports provide a foundation for understanding how ZIKV interacts with placental cells, assessment of the virus produced by these cells, and the ability of the virions produced to infect new cells, was not defined. We demonstrated that PRVABC59 was able to infect HC and trophoblasts as quantified by RT-PCR (Figure 1).

We further demonstrated that blockade of Jak ½ signaling with a supraphysiological concentration (1 μM; approximately 1 log above the steady-state plasma concentrations achieved for all FDA-approved doses of ruxolitinib [22]) of the Jak inhibitor ruxolitinib resulted in 24-fold to > 500-fold more virus produced in these cells compared with infections in the absence of ruxolitinib (RT-PCR; Figure 1). It is likely that the immunological antiviral profile of trophoblasts previously reported in the presence of Zika virus [8] could be responsible for the markedly increased viral replication (> 500-fold) conferred in trophoblasts by ruxolitinib. The natural IFN-based antiviral profile is robust in trophoblast cells [8], and it is reported to confer a natural block on Zika virus replication in these cells. Blockade of this IFN-based antiviral effect reported in trophoblasts by ruxolitinib likely confers a robust increase in viral replication in the presence of 10 μM ruxolitinib.

To understand the implications of Jak ½ inhibition on virus production and the cellular milieu that may impact MTCT, we sought to observe markers associated with activation and MTCT, DC-SIGN and HLA-DR [19, 31]. The marker DC-SIGN in particular, is increased during pregnancy in the placenta and its expression has been correlated with increased rates of vertical transmission of viruses, including HIV, which underscores its potential as a modality for MTCT [31]. DC-SIGN and HLA-DR were significantly (*P* < 0.05, Figure 2) increased compared with uninfected controls for infection with PRVABC59, and addition of ruxolitinib significantly increased both markers compared with infected, non-ruxolitinib treated HC *P* < 0.05, Figure 2). These data demonstrated that infection of HC may confer activation that could stimulate MTCT, and that Jak/STAT blockade further amplifies this axis, which emphasizes the role of Jak-STAT signaling in modulating the infection, activation, and potential MTCT governed by these events.

Ruxolitinib is an anti-inflammatory agent under clinical investigation for use in 43 different inflammatory disorders in humans (Jakafi.com). In addition, ruxolitinib is a potent inhibitor *in vivo, ex vivo*, and *in vitro* of inflammatory cytokines IL-6, D-dimer, TNF-α, IFN-α/β, C-reactive protein (CRP), and IL-1α/β (Jakafi.com). This panel of inflammatory cytokines are blocked by ruxolitinib independently from the clinical indication responsible for conferring this increase in inflammatory markers.

Because recent reports have demonstrated that ZIKV can infect primary neuronal cells, and ZIKV infection of neurospheres and the CNS is a major hallmark of brain deformities, abnormalities, microcephaly, or other detrimental effects [2, 3, 6, 32, 33], we sought to determine if Jak-STAT pathway inhibition may be modulating infection in human neuroblastoma cells. We demonstrated that supraphysiological concentrations of ruxolitinib (1 and 10 μM) in neuroblastoma cells significantly (*P* < 0.05, Figure 4) increased 4G2^+^ cells compared with infections in the absence of ruxolitinib. A mechanism that flaviviruses employ to establish primary infection, and to evade the innate immune response and subsequent triggering of adaptive immunity is blockade of IFN production by inhibition of the Jak-STAT pathway [9, 12, 14, 15, 18]. It is possible that further blocking of IFN-α/β by ruxolitinib, and selective Jak/STAT inhibition by this drug may promote infection. Together, these data confirm that the Jak-STAT pathway is a key effector in permissiveness of clinically relevant target cells.

Supernatants from trophoblasts and HC infected with ZIKV PRVABC59 were unable to induce CPE in Vero cells (which are IFN deficient), and 1 μM of ruxolitinib, a supra-physiological concentration of drug added to HC or trophoblast cultures during infections, resulted in production of virions that were able to confer virally induced CPE in uninfected Vero cells using HC, but not trophoblast supernatants (Figure 3). We chose a supraphysiological concentration of the drug to ensure complete blockade of Jak ½ signaling so that the impact of full blockade of this pathway on ZIKV could be evaluated. At physiological concentrations of ruxolitinib, some Jak ½ signaling still occurs, which allows functional adaptive and innate immunity *in vivo*, therefore higher concentrations were used for this study [22]. We further demonstrated that antiviral agents 2′-C-MeC and NHC at various concentrations added to HC or trophoblast cultures in the presence of 1 μM ruxolitinib produced a concentration-dependent inhibition of virus (Figure 3); this was quantified by extracellular transfer of supernatants collected from HC or trophoblasts and subsequent transfer and calculation of EC_50/90_ in uninfected Vero cells. Reduction in virus produced by HC or trophoblasts in the absence of ruxolitinib by antiviral agents could not be quantified in this manner, because supernatants produced from these cells did not confer virally induced CPE in uninfected Vero cells (Figure 3), suggesting that the virus produced may not be replication competent.

To better understand if the Jak ½ block results in differences in viral integrity produced in HC and trophoblasts, negative-stain electron microscopy was performed with virions produced in these cells with and without 1 μM ruxolitinib, compared with virions from Vero cells (which are known to produce replication-competent virions [27, 34]. Electron microscopy demonstrated that virions from HC or trophoblasts infected in the absence of ruxolitinib contained a “thin coat”, similar to immature virions observed for other flaviviruses including DENV [35–37]. In contrast, virions produced from Vero cells contained a “thick coat” representative of the mature virion structure reported for ZIKV and for other flaviviruses [35–37]. Addition of 1 μM ruxolitinib to HC and trophoblasts during infection resulted in production of thick coat virions whose appearance resembled that reported for mature ZIKV [38]. Although virions from trophoblasts produced in the presence of ruxolitinib appear mature, these supernatants were not able to induce viral CPE in the highly permissive Vero cell line. We observed by EM that very few virions were produced from trophoblasts, and previous reports have demonstrated that infection in trophoblasts was markedly lower than that observed in HC [25]. Therefore, it is possible that the addition of ruxolitinib allows trophoblasts to produce mature virions, but that the amount of virions produced is too low to confer productive infection in uninfected cells. The addition of ruxolitinib resulted in production of a thick coat and a structure that is akin to that reported for mature ZIKV virions. These data suggest that the amount of mature virions produced in HC, but not trophoblasts, in the presence of ruxolitinib are great enough to confer infection in naive cells. These data also emphasize that virus produced in HC and trophoblasts in the absence of ruxolitinib appear to be immature, which could account for observed lack of replication competence. Production of mature proteins requires furin and other maturation-associated host factors that are modulated by the activation state of cells [13, 34, 39] and also linked to differential cellular metabolism/activation events [13, 14, 34, 40].

Therefore, it is possible that the down-regulation of activation that has been reported for ruxolitinib, as well as its blockade of IFN-α/β production (which is a noted antiviral response to flaviviruses [8–10, 12, 25, 41]), could be in part responsible for the observed differences in virions produced and their ability to infect naive Vero cells. Specifically, this mechanism may be responsible for the finding that only virus produced in the presence of ruxolitinib present the morphology of mature virions (observed in HC and trophoblasts), and are capable of conferring virus-induced CPE in naive Vero cells (observed in HC).

Together, these findings demonstrate that the Jak-STAT pathway is a key gatekeeper in production of mature virions in clinically relevant cells including HC and trophoblasts. Clearly elucidating the key factors involved in ZIKV infection, the production of mature, replication- competent virions, and activation events that are implicated in MTCT provide a foundation for the design of safe, specific agents that can target these events.
